# Incidence and management of mallet finger in Dutch primary care: a cohort study

**DOI:** 10.3399/BJGPO.2023.0040

**Published:** 2024-01-24

**Authors:** Patrick Krastman, Evelien de Schepper, Patrick Bindels, Sita Bierma-Zeinstra, Gerald Kraan, Jos Runhaar

**Affiliations:** 1 Department of General Practice, Erasmus MC University Medical Center Rotterdam, Rotterdam, The Netherlands; 2 Department of Orthopedics & Sports Medicine, Erasmus MC University Medical Center Rotterdam, Rotterdam, The Netherlands; 3 Department of Orthopedic Surgery, Reinier de Graaf Groep, Delft, The Netherlands

**Keywords:** incidence, management, mallet finger, general practice, primary health care

## Abstract

**Background:**

A mallet finger (MF) is diagnosed clinically and can be managed in primary care. The actual incidence of MF and how it is managed in primary care is unknown.

**Aim:**

To determine the incidence of MF in primary care and to obtain estimates for the proportions of osseous and tendon MF. An additional aim was to gain insight into the management of patients diagnosed with MF in primary care.

**Design & setting:**

A cohort study using a healthcare registration database from general practice in the Netherlands.

**Method:**

Patients aged ≥18 years with a new diagnosis of MF from 1 January 2015–31 December 2019 were selected using a search algorithm based on International Classification of Primary Care (ICPC) coding.

**Results:**

In total, 161 cases of MF were identified. The mean incidence was 0.58 per 1000 person–years. A radiograph was taken in 58% (*n* = 93) of cases; 23% (*n* = 37) of cases had an osseous MF. The most applied strategies were referral to secondary care (45%) or conservative treatment in GP practice (43%). Overall, 7% were referred to a paramedical professional.

**Conclusion:**

On average, a Dutch GP assesses ≥1 patient with MF per year. Since only a minimal number of patients required surgical treatment and a limited number of GPs requested radiography, the recommendation in the guidelines to perform radiography in all patients with MF should potentially be reconsidered. The purpose of requesting radiographs should not be to distinguish between a tendinogenic or osseous MF, but to assess whether there is a possible indication for surgery.

## How this fits in

Mallet finger (MF) is a traumatic finger injury of which the diagnosis is mainly made on clinical signs and symptoms. To the authors’ knowledge, this is the first study that provides an insight into the incidence of MFs in primary care, as well as the applied management strategies by GPs. On average, a Dutch GP assesses ≥1 patient with a MF per year. The study found a discrepancy between guideline-recommended treatment strategies and the applied management by GPs. There was less use of imaging and greater referral to secondary care compared with guideline recommendations. Since only a minimal number of patients required surgical treatment and radiography requests were limited, the recommendation in the guidelines to perform radiography in all patients with MF should potentially be reconsidered.

## Introduction

MF results following forceful flexion of an extended distal phalanx, causing extensor tendon disruption, either isolated or in combination with a distal phalanx avulsion fracture.^1–4^ The diagnosis of MF is mainly made on clinical signs and symptoms. Based on current literature and guidelines, radiography should always be performed to differentiate between an osseous mallet injury or a tendinous one and assessment of volar subluxation of the distal phalanx.^5,6^ A tendinous MF is more common than an osseous MF.^7^

Despite the feasibility of MF diagnosis and treatment in primary care, the incidence figures and management of MF in primary care is still unknown.^6^

In primary and secondary care, the treatment of a tendinogenic MF or an osseous MF is mainly conservative with continual splinting or casting of the distal interphalangea (DIP) joint in extension for 6–8 weeks, followed by a programme to gradually increase range of motion.^1,6,8^ The treatment of most MFs can be performed by GPs and hand therapists in primary care.^6,9^ Consensus is lacking on the indications for the surgical treatment of an osseous MF.^10^

Currently, there is a clear absence in knowledge on the incidence and management of MF in primary care. The objectives of the study were as follows: 1) to determine the incidence of MF in primary care; 2) to obtain estimates for the proportions of osseous and tendon MF; and 3) to gain insight into the management strategies of patients diagnosed with MF in primary care.

## Method

### Study design

A cohort study using the Rijnmond Primary Care database (RPCD) was conducted. The RPCD is a region-specific product of the Integrated Primary Care Information (IPCI) database, supervised by the Department of General Practice of the Erasmus University Medical Center (Erasmus MC), Rotterdam, the Netherlands. More information about the IPCI database has been described in detail elsewhere.^11^ In short, the RPCD is a longitudinal observational dynamic database containing medical records of >200 000 primary care patients in the larger Rotterdam area.^12^ All citizens in the Netherlands are obliged to register with a GP practice. A Dutch GP forms the first point of care for all health-related problems and acts as a gatekeeper.^13,14^ These pseudonymised medical records contain demographics, medical notes (free text), diagnoses (including International Classification of Primary Care [ICPC] codes), referrals, imaging results, and specialists’ letters that are routinely collected by GPs. The database included approximately 25% of the population of the area of Rotterdam, equally distributed across the region and including neighbourhoods with different socioeconomic and migration levels.

### Study cohort

The study population consisted of adults aged ≥18 years with a new episode of MF between 1 January 2015 and 31 December 2019. The diagnosis was considered new if the patient had not been diagnosed with MF in the preceding 12 months. Patients could be included in the study population more than once if there was >12 months between the initial diagnosis and subsequent consultations for MF. Diagnoses of MF made at the out-of-hours GP service are processed in the GP information system, and are therefore also included in the database of the RGPD. Diagnoses of MF were identified using ICPC coding and with supporting keywords in the free text.^15^ Patients were considered a ‘potential MF case’ if they received the ICPC code L98.01 (mallet finger) or if they received the ICPC code L12 (hand, finger symptoms, or complaint), L74.01 (fracture phalanges hand), or L81 (other musculoskeletal injuries), in combination with the (Dutch) words ‘mallet’, ‘phala’, ‘fala’, ‘digit’, ‘pees (tendon)’, ‘extensor’, or ‘gewricht (joint)’ in the free text. This algorithm excluded cases in which these words were combined with terms of negation (for example, not or no).

### Data extraction

To obtain the positive predictive value (PPV) after running the search algorithm, 200 random medical files from potential cases throughout the study period were manually reviewed (PK). These files were examined from the consultation date of the initial diagnosis up to 6 months after the first consultation. Potential cases were a true MF case if the GP defined the consultation as an MF injury. Unclear cases were also assessed by a hand surgeon (GK), and final decisions were based on consensus. For each true case in the 200 random samples, information on year of birth, sex, the affected finger, trauma mechanism, whether a radiograph was taken, osseous or tendon MF, and the number of consultations with the GP for one episode of complaints were extracted (PK). The following management strategies were registered for all patients with MF who were initially diagnosed and treated by their GP: policy by GP (conservative treatment in GP practice); referral to a secondary care specialist (trauma surgeon, orthopaedic surgeon, or plastic surgeon); referral to a paramedical professional (hand therapist, physiotherapist, or ergo therapist); and treatment (conservative or surgically) by secondary care specialist. Telephone consultations were counted as consultations, provided that the GP registered a contact with the patient and discussed the diagnosis or management of MF.

### Statistics

Incidence numbers per study year, as obtained by the search algorithm, were multiplied with the estimated annual PPV to obtain yearly incidence estimates. The 95% confidence intervals (CIs) were calculated from the incidence, using Poisson distribution. The annual incidence of MF per 1000 person–years was also subdivided for males and females, and for age categories 18–40 years, 41–60 years, and ≥61 years. Descriptive data for the identified cases (age, affected finger, number of consultations, trauma mechanisms, number of radiographs, and referral) were described as numbers (percentages), medians (interquartile range; IQR), and means (standard deviation; SD). All analyses were performed using IBM SPSS Statistics (version 27.0). R Studio was used for Poisson distributions.

## Results

### Study population

**Figure 1.**
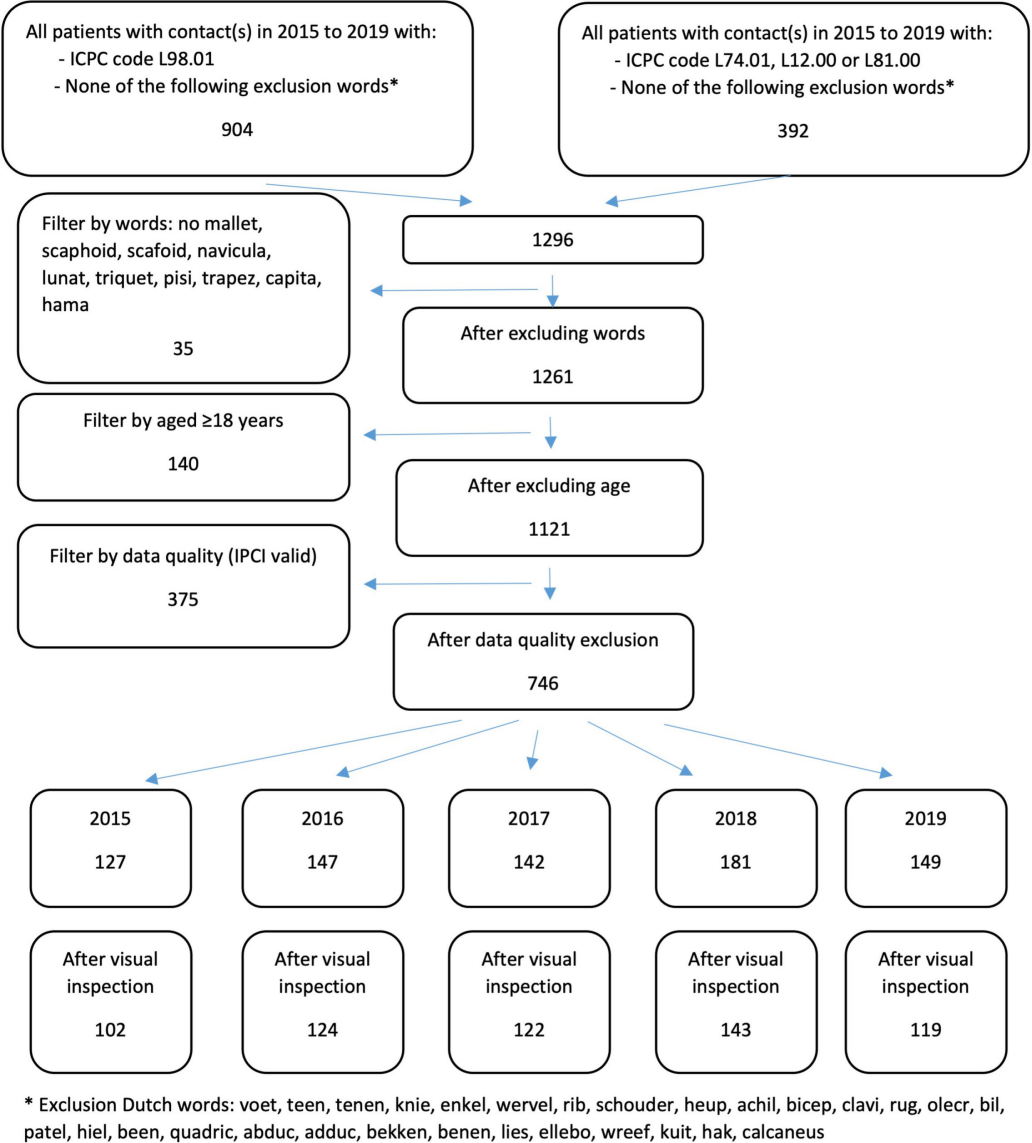
Flow diagram for the study population. ICPC = International Classification of Primary Care. IPCI = Integrated Primary Care Information.

The flow diagram for the study population is presented in Figure 1.

**Figure 2.**
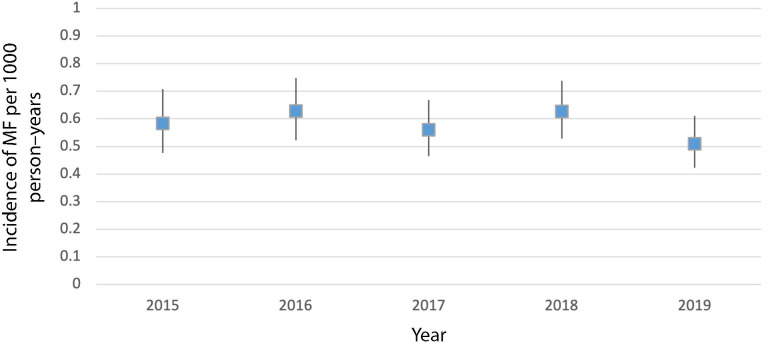
Annual incidence of mallet finger (MF) per 1000 person–years

The number of patients aged ≥18 years in the RPCD ranged from 185 093 in 2015 to 254 345 in 2019. The query showed 127, 147, 142, 181, and 149 unique potential cases of MF from 2015–2019, respectively (Figure 1). Of the 200 potential MF cases assessed over the entire study period, 161 were a confirmed case (PPV 0.81). Using the annual PPV, the number of patients per year with an actual MF were 102, 124, 122, 143, and 119 from 2015–2019, respectively (Figure 1). The annual incidence of MF per 1000 person–years is displayed in Figure 2.

**Figure 3.**
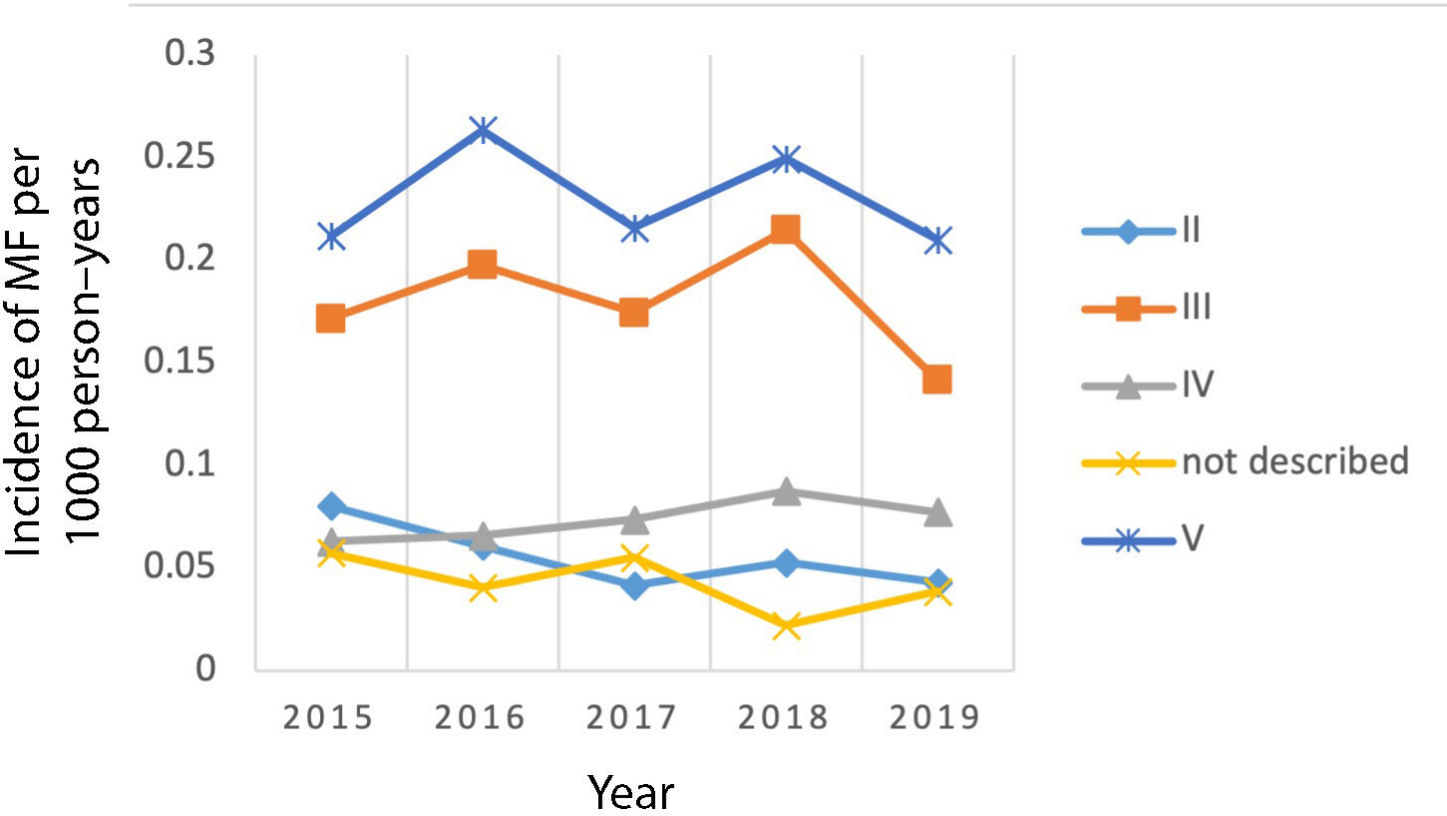
Annual incidence of mallet finger per 1000 person–years, stratified per finger

The mean incidence over the study period was 0.58 (95% CI = 0.48 to 0.69) per 1000 person–years and ranged from 0.51 in 2019 to 0.63 in 2016 and 2018 (Figure 2). The mean age at diagnosis was 53 years (SD 15.4, range 18–91 years) (data not shown). The fingers affected are displayed in Figure 3. Digit III and V were the most common injured fingers. MF of the thumb was not observed during the study period. Of note, some of the medical records did not describe which finger was affected (*n* = 44).

**Figure 4.**
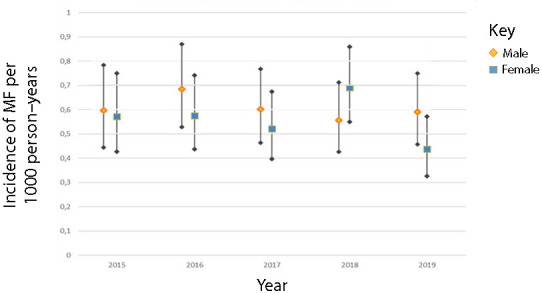
Annual incidence of mallet finger per 1000 person–years for males and females

Males had a mean incidence rate of 0.60 (95% CI =0.46 to 0.77) per 1000 person–years and females had a mean incidence rate of 0.56 (95% CI = 0.43 to 0.72) per 1000 person–years (see Figure 4). Peak incidence was between 41 and 60 years for males and 61 and 80 years for females (data not shown).

### Management

Of the patients with MF, 93% (*n* = 150) were initially diagnosed and managed by the GP, with the remaining patients diagnosed and managed at the emergency department of a hospital (data not shown). For the patients initially evaluated in primary care, the number of consultations with the GP had a median of two (IQR 1–2). The trauma mechanisms, total and stratified for age categories, are shown in Table 1. Household activities (16%) and leisure (18%) were the most noted causes for MF. In 38% of cases it was not possible to determine the trauma mechanism.

**Table 1.** Trauma mechanism for the development of mallet finger, stratified by age categories

Trauma mechanism	Patients aged 18–40 years,*n* (%)	Patients aged 41–60 years,*n* (%)	Patients aged ≥61 years,*n* (%)	Total,*n* (%)
Household activity	5 (14)	8 (11)	13 (24)	26 (16)
Leisure	6 (17)	11 (16)	12 (22)	29 (18)
Other	3 (8)	10 (14)	4 (7)	17 (11)
Sport	8 (22)	5 (7)	4 (7)	17 (11)
Work	3 (8)	8 (11)	0 (0)	11 (7)
Not described	11 (31)	29 (41)	21 (39)	61 (38)
Total	36 (100)	71 (100)	54 (100)	161 (100)

In 58% (*n* = 93) of cases, a radiograph was taken at or directly after the first presentation. Among these, 23% (*n* = 37) of cases had an osseous MF confirmed by radiography, 35% (*n* = 56) had a tendon MF, and for 42% (*n* = 68) no distinction could be made (data not shown).

Referral to a secondary care specialist after the initial assessment by the GP was the most applied strategy (45%), followed by conservative treatment in general practice (43%), and referral to a paramedical professional (11%) (Table 2). Of the patients in whom the GP initially started conservative treatment in general practice, during follow up, six (8%) patients were referred to a secondary care specialist and five (7%) to a paramedic professional. The minimum and maximum number of weeks after which the patient was referred were 1–14 weeks (mean 8.5; SD 4.97) for secondary care specialist and 6–12 weeks (mean 8.4; SD 2.6) for paramedical professionals, respectively (data not shown).

**Table 2.** Management of mallet finger cases at initial consultation, adjustment to initial management strategies, and final management strategies including adjustments

Management	Management strategy at initial consultation, *n* (%)	Additional management strategy, *n* (%)	Final management strategy, *n* (%)
Conservative treatment in general practice	75 (50)	0 (0)	64 (43)
Referral to secondary care	62 (41)	6 (55)	68 (45)
Avulsion fracture	25 (17)	0 (0)	25 (17)
Not described	31 (21)	0 (0)	31 (21)
Other reason	6 (4)	3 (27)	9 (6)
Delayed recovery	0 (0)	3 (27)	3 (2)
Referral to a paramedic professional	6 (4)	5 (45)	11 (7)
Not described	7 (5)	0 (0)	0 (0)
Total	150	11	150

Overall, 2% (*n* = 4/161) of patients with MF underwent surgery within 6 months of their initial presentation (data not shown). The mechanism of origin of the MF involved a high-energy trauma in two patients (finger caught between a trailer and bus, and falling from a height), and the trauma mechanism was unknown in the other two patients. Three patients had an intra-articular avulsion fracture and in the fourth patient an MF without a fracture (tenodermodesis) was diagnosed.

## Discussion

### Summary

To the best of the authors' knowledge, this is the first study to determine the mean incidence of MF in primary care, which was 0.58 per 1000 person–years. Indicating that, on average, a GP with an average practice size of 2100 patients assesses ≥1 patient with MF per year. In the majority of patients (58%), GPs requested radiography. Surgical treatment was required in only 2% of patients. Based on our study, the recommendation in the guidelines to perform radiography in all patients with MF should potentially be reconsidered. Referral to secondary care and conservative treatment in the GP practice were the dominant management strategies.

### Strengths and limitations

The RPCD population is representative for the general Dutch population in terms of age and sex (see Supplementary Information S1). The geographical spread is limited, but GP practices are located both in urban and non-urban areas. Despite the limited geographical spread, we do not expect that this will have influenced our results, because MF injuries are frequently sustained during either work or participation in sports, and they are most common in adult males; all of which are well represented within the studied population.^16^ Data were unavailable for type of health insurance or socio-economic status of patients in RPCD.^11^ The use of registration data can been seen as a strength of this study; GPs were not influenced in their management approach, and it can therefore be assumed that the study provided a true representation of the management of MF performed by GPs. Despite all Dutch citizens being registered at and managed by GPs, it is possible that patients with MF have been diagnosed and managed by a paramedical professional without first being assessed by a GP, possibly resulting in a higher incidence rate of MF in primary care than described in this study. In the Netherlands, patients have direct access to a paramedical professional without requiring a referral from the GP. Selection bias may have occurred in this study owing to the dependence on 1) the diagnostic accuracy of the GP in making the diagnosis of MF, and 2) (in)adequate reporting in the medical file by the GP.^17^ Since this study was dependent on GP medical records, the incidence can be considered as the incidence of registered consultations only. To limit the possible underestimation of the overall incidence owing to limited medical notes or non-uniform ICPC coding, multiple ICPC codes and free-text terms were used to identify patients with MF. Another limitation could be that management results of medical specialists are only available if the specialist sends communication to the GP and the GP has adequately processed the content of the letter. This could have given a slight underestimation of the real number of patients who received imaging and a certain (surgical) treatment in the hospital.

### Comparison with existing literature

A mean incidence of 0.58 (95% CI = 0.48 to 0.69) per 1000 persons–years was found in our study. Peak incidence was between aged 41 and 60 years for males and 61 and 80 years for females. Currently, to the authors’ knowledge, literature regarding the incidence and management of MF in primary care is lacking. One large retrospective study of MF at a UK hospital’s emergency department reported an incidence of 9.9 per 100 000 patient–years.^18^ This study also described that MF was most common in males aged 25–55 years and that the incidence began to drop after the fifth decade, at which it becomes equal to the incidence in females. Based on the study of Clayton and Court-Brown, one could hypothesise that males in the age category of 25–55 years with MF are more likely to be assessed by the emergency department than by a GP.^18^ In the current study, the incidence of MF was six times higher than was reported at the emergency department. Potentially, most patients with MF, both in the Netherlands and in the UK, first report to a GP, who can refer these patients to secondary care, if necessary, without attending an emergency department.

Based on the results of the current study, it seemed that the GPs did not adhere to the current literature and several guidelines, as not all patients with MF underwent guideline-recommended imaging.^5,6,19–21^ According to current literature and (inter)national guidelines, radiography should always be recommended to differentiate between an osseous mallet injury (assessment articular surface) and a tendinous one, and provide assessment of volar subluxation of the distal phalanx. Remarkably, in the current study, imaging was only used in a limited number of all cases (58%).^5,6,19,20^ Imaging is advised in the guidelines because surgery should be considered for patients with an osseous MF with an avulsion fragment larger than 30%–40% of the joint surface and/or a volar subluxation of the distal phalanx.^6,19–21^

In a long-term follow-up study, complications of treatment in 123 operatively and non-operatively treated MFs were reviewed.^22^ In the 84 digits treated with splints, there was a 45% rate of complication (for example, skin, transverse nail grooves, and pain), which were almost always transient, and the complication often depended on the splint type selected. For the 45 surgically treated digits, the complication rate was 53% (for example, nail deformities, joint incongruities, infection, pin or pull-out wire failure, and radial or ulnar prominence or deviation of the DIP joint), with 76% of these complications still being present after a mean follow-up of 38 months.^22^ Redislocation occurred in seven digits (16%), requiring additional surgery.^22^ Based on these results, the authors advocated for splinting in the treatment of almost all MF injuries.

Another long-term follow-up study, focusing on osteoarthritis (OA) of the DIP joint after MF fracture, showed that the rate of radiological OA development after MF fracture was almost equal to the natural degenerative process in the DIP joint in the absence of prior MF fracture and was accompanied by a decrease in range of motion of the DIP joint, which did not clinically affect patient-related outcome measures.^23^ Therefore, conservative treatment of MF was advocated. The current study showed that only 2% of patients eventually had to undergo surgery. Based on this knowledge, the purpose of requesting radiographs should not be to distinguish between a tendinogenic or osseous MF, but to assess whether there is a possible indication for surgery. Based on results from the literature described above and the low percentage of patients who underwent surgical intervention in the current study, the guideline recommendations to perform radiography in all patients with MF should potentially be reconsidered. Requesting radiographs should only be recommended if the MF has developed after high-energy trauma (for example, crush injuries).

Based on current (inter)national guidelines, clinicians are advised to assess patients with MF on a regular basis, and to evaluate the effect of conservative therapy or to consider hand therapy to accurately guide the non-surgical splinting and postoperative treatment of osseous MFs.^6,17,19,21^ Nevertheless, in the current study GPs treated patients with MF differently, given the limited number of GP consultations (median 2), few referrals to a paramedic professional (7%), and many referrals to a secondary care specialist (45%).

### Implications for research and practice

This study found that, in the Netherlands, a GP sees, on average, one patient per year with MF, and 2% of patients with MF undergo surgery in the first 6 months after initial presentation. Based on the current literature and guidelines, radiography is always advised for a patient with MF. Future research should investigate the added value of imaging in all patients with MF in primary care, given the low number of patients who underwent surgery. There was a discrepancy between the current guidelines and how GPs treated patients with MF in clinical practice. Future research should investigate why GPs do not refer all patients with MF for radiography and why they refer more often to a secondary care specialist rather than to a paramedical professional.

## Data

Data are not publicly available.
